# The neonatal onset diabetes mellitus of Chinese neonate with congenital generalized lipodystrophy 2: a case report

**DOI:** 10.1186/s12902-022-00992-x

**Published:** 2022-03-29

**Authors:** Yuan Yang, Li Ma, Jingjing Sun, Xiaohui Gong, Cheng Cai, Wenchao Hong

**Affiliations:** Department of Neonatology, Shanghai Children’s Hospital, Shanghai Jiao Tong University, Shanghai, 200062 China

**Keywords:** Congenital generalized lipodystrophy, Neonate, Metabolic disorders, BSCL2, Case report

## Abstract

**Background:**

Congenital generalized lipodystrophy (CGL) is a clinically heterogeneous disorder characterized by near total absence of adipose tissue along with metabolic complications. Diabetes mellitus developed from CGL usually present between ages 15 and 20 years, and there are few reports in neonate.

**Case presentation:**

In this report, we described a rare clinical presentation of CGL in a 12-day-old Chinese female neonates with hyperglycemia, hyperlipidemia, and subsequently appeared diabetes, hepatomegaly and fatty liver. The two clinical-exome sequencing identified heterozygous null mutations (c.793C > T and c.565G > T) in BSCL2 gene which was inherited from father and mother respectively. To date, it was the firstly reported CGL patient with neonatal onset diabetes. The neonate was treated with antibiotic, insulin and deeply hydrolyzed formula milk to significantly decrease FBG and serum trigylcerides levels.

**Conclusions:**

Our case report analyzes the causes of early onset diabetes may relate with the locus of BSCL2 gene mutations and infection induction. It also suggests the importance of early identification, genetic analysis, and symptomatic treatment in the CGL, which are essential for improving the prognosis of children.

## Background

Congenital generalized lipodystrophy (CGL) is a rare autosomal recessive inherited diseases characterized by absence of adipose tissue, skeletal muscle hypertrophy and metabolic complications related to insulin resistance (IR) [1]. About 25–35% of CGL patients with IR develop diabetes between ages of 15 and 20 years [2]. CGL is classified into four different subtypes according to pathogenic genes, all of which play a key role in the synthesis of phospholipids and triglycerides. CGL2 is the most severe form of generalized lipodystrophy, caused by mutations in *BSCL2* gene. In this report, we described a case of CGL2 in a 12-day-old Chinese female neonate presented with typical hypertriglyceridemia and diabetes, which are very rare at this age. Furthermore, we performed a Clinical exome sequencing (CES) that revealed two heterozygous null mutations in the BSCL2 gene in this patient.

## Case presentation

**Fig. 1:**
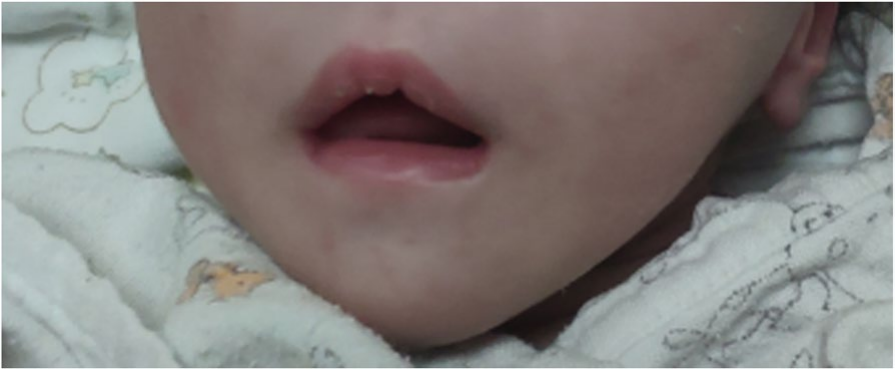
Patient at the age of 3 weeks: clinical photograph revealing lipoatrophy phenotype

**Fig. 2:**
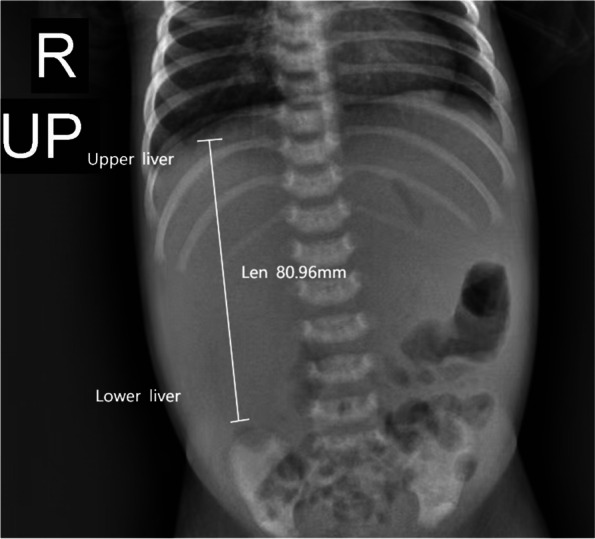
Abdominal X - ray: liver enlargement

A 12-day-old female neonate was admitted to local hospital for anorexia, lethargy and fever. Biochemical tests showed the fasting blood-glucose (FBG) level was 20.3 mmol/L and triglyceride (TG) was 16.71 mmol/L. Although treated with antibiotics and insulin intravenous infusion, her symptoms did not improve. Therefore, she was transferred to the neonatal intensive care unit (NICU) of our hospital 4 day later. The newborn’s mother had hyperthyroidism during pregnancy. She was delivered naturally at 41 weeks gestational age with turbidity amniotic fluid and slightly short umbilical cord. Her APGAR scores at 1 min after birth was 7 and at 5 min was 9. Her birth weight was 3260 g. She was the first child of non-consanguineous parents without any family history of hereditary diseases. On admission to our NICU, the weight of the patient was 3490 g, height was 50 cm and occipitofrontal circumference was 34.5 cm. During the physical examination, we found her lipoatrophy phenotype (Fig. 1) and the liver palpated 8 cm below the right middle clavicle (Fig. 2).

Then she was given a series of related laboratory tests on the day of hospitalization. The blood routine analysis showed the white blood cells (WBC) count of 8.36 × 10^9^/L and the C reactive protein (CRP) concentration count of 13 mg/L. Blood culture was negative. Biochemical tests showed no abnormalities in the liver and kidney function, FBG level was 17.8 mmol/L, serum sodium was 129 mmol/L, TG was 42 mmol/L. The level of C peptide was 4.60 nmol/L and the level of insulin was 520.2 pmol/L. Thyroid function examination did not show significant abnormality. Abdominal CT indicated liver enlargement and hepatic adipose infiltration. Cardiac ultrasound showed a atrial septal defect of 1.6 mm.

**Fig. 3:**
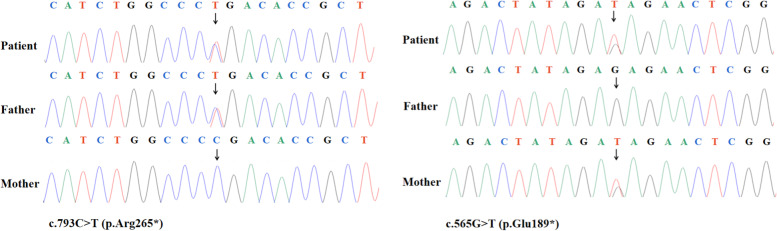
Genomic sequence of the patient. Genetic analysis revealed two heterozygous null mutations in the patient’s BSCL2 gene, which was respectively detected from her father and mother. The arrows indicated the mutation site

We suspected a clinically diagnosed of GCL when early diabetes, severe hypertriglyceridemia, and hepatosplenomegaly occur [1]. After obtaining the informed consent of the parents, we performed trio clinical exome sequencing on the peripheral blood of the newborn and her parents. Genetic analysis revealed two heterozygous nonsense mutations in the patient’s *BSCL2* gene [c.793C > T (p.Arg265*), c.565G > T (p.Glu189*)] inherited from the father and mother respectively. Sanger sequencing verified this conclusion (Fig. 3). Both mutations have been recorded as pathogenic mutation in the Human Gene Mutation Database (HGMD) and ClinVar database.

After receiving antibiotics and insulin treatment, her FBG was decreased to 6 mmol/L without insulin. She was gradually fed with deeply hydrolyzed formula (containing 40% medium chain triglycerides). The laboratory tests show TG was reduced to 17.57 mmol/L 1 week later. However, her parents decided to end all treatment and released from hospital after 2 weeks of in-hospitalization. We followed up the patient by phone when she was 5 months old. The patient’s weight was 6000 g by fed with common formula. Her FBG was maintained around 7 mmol/L without insulin treatment. Biochemical blood test results suggest the presence of abnormal liver function. Unfortunately, parents did not follow our advice to follow up regularly at the hospital.

## Discussion and conclusions

In this case report, we summarized the diagnosis and treatment process of a 12-day-old Chinese girl with CGL2. Berardinelli described the first characterization of this syndrome in 1954 and confirmed by Seip in 1959 [3, 4]. The diagnostic presentation of our patient with lipoatrophy phenotype, early diabetes severe hypertriglyceridemia, and hepatosplenomegaly was suggestive of CGL and latter was verified by genetic diagnosis by clinical-exome sequencing. Mutations in different genes cause four different subtypes of CGL [5]. The BSCL2 gene locates on chromosome 11q13 and can encodes a 398 amino acid transmembrane protein encoding seipin protein. Seipin is a transmembrane protein concentrated in the endoplasmic reticulum and plays an important role in lipid droplet assembly and adipocyte differentiation [6]. Seipin deficiency can leads to lipodystrophy by mediating abnormal differentiation and development of adipose tissue [7–9].

Due to the abnormal development of adipose tissue and excessive accumulation of triglycerides, many severe clinical complications of CGL occur [10, 11]. These clinical complications such as hyperglycemia, hyperlipidemia, diabetes, hepatomegaly and fatty liver were observed in our patients. Other associated anomalies include almost complete absence of adipose tissue, skeletal muscle hypertrophy and mild intellectual impairment, which were not all noticed in our neonate [10]. Although metabolic abnormalities such as hyperinsulinemia and IR can occur early in the disease, 25–35% of CGL patients with IR develop diabetes between ages of 15 and 20 years [2]. A similar report was published by Van Maldergem et al. in which the authors reported a CGL patient diagnosed at 4 months, diabetes was diagnosed by 15 years [12]. Similarly, another report found a child who was diagnosed at 9 months of age with CGL and also developed diabetes 13 years later [10]. Finally, a series of case reports published by Indumathi et al. and Friguls et al., found that children with CGL2 developed diabetes at the age of 4–5 months [11, 13, 14]. In our case study, our neonate developed diabetes at a very early age of 12 days and is the youngest reported CGL patient with diabetes so far. Early diabetes can increase the risk of developing acute infections and cardiovascular diseases and lead to poor prognosis of the nervous system.

We tried to found the causes of early onset diabetes from genetic analysis. In our report, patient’s BSCL2 gene carried heterozygous mutations and the mutations were both classified as pathogenic. Compared with other types of CGL, patients with BSCL2 mutations had an earlier onset of diabetes [15]. Through literature review, we found and compared clinical features of other cases sharing hemizygous mutation with ours. A similar report found an identical mutation [c.565G > T (p.Glu189*)] in a 7-year-old child and a 4-month-old infant [13, 15]. Both of whom were found to be homozygous for this mutation and presented with hyperinsulinemia, early onset diabetes, hypertriglyceridemia and cardiac phenotype. Early-onset diabetes and hypertriglyceridemia were main characteristics in our case of study (Table 1). It may be associated with the substitution of glutamic acid at codon 189 with a stop codon. So far, the cases in our report have not had cardiac and neurological abnormalities. Another mutation [c.793C > T (p.Arg265*)] in our case was firstly reported by Guillen-Navarro et al. [16] as c.985C > T. They reported six children with the same c.985C > T mutation including homozygous and heterozygous mutation. Children with homozygous mutations showed mild clinical features of CGL2 yet suffered from progressive encephalopathy from 2-year-old and death at the age of 6–8 years. The patients with heterozygous mutations showed typical CGL phenotypes in addition to similar neurological processes to homozygous patients. This mutation can result in complete jump of exon 7, altered reading frames, early termination and abnormal protein expression which eventually leads to extremely severe nervous system syndrome. Our patient with heterozygous mutations showed typical CGL phenotypes shortly after birth and with no neurological symptoms so far. Given her genetic background and relevant reports, clinicians should pay more attention to neurodevelopment and cardiac abnormalities. She may have some of the above complications during growth. Unfortunately, parents did not follow our advice to follow up regularly at the hospital.

**Table 1:** Clinical features of three patients with similar mutant of BSCL2

Clinical features	Patient 1	Patient 2	Patient 3
Sex	Woman	Woman	Man
Age of onset	12-day-old	7-year-old	4-month-old
Physical examination			
Skin	Normal	Acanthosis nigricans, Hirsutism	Hirsutism
Fat	Face lipoatrophy	General lipoatrophy	General lipoatrophy
Muscle	Normal	Muscular hypertrophy	Muscular hypertrophy
Acra	Normal	Acromegaloid features	Acromegaloid features
Heart	Atrial septal defect	Cardiac murmur	Cardiac murmur
Liver	Hepatomegaly	Hepatomegaly	Hepatomegaly
Other characteristics	Simian line	Crassitude of the penis	
Laboratory examinations	Hypertriglyceridemia	Hypertriglyceridemia	Hypertriglyceridemia
	Diabetes	Diabetes	Diabetes
	Hepatic adipose infiltration	Hyperinsulinemia	Hyperinsulinemia
	Liver dysfunction	hepatocirrhosis	Hepatic adipose infiltration
		Liver dysfunction	Liver dysfunction
		Left ventricle hypertrophy	Pancreatitis
		Mild mental retardation	Severe obstructive and asymmetrical septal hypertrophic cardiomyopathy
Gene analysis	c.793C > T(p.Arg265*)	c.565G > T(p.Glu189*)	c.565G > T(p.Glu189*)
	c.565G > T(p.Glu189*)	c.565G > T(p.Glu189*)	c.565G > T(p.Glu189*)

Patient 1, the patient in our case; Patient 2, the patient reported by Jin et al. (15); Patient 3, the patient reported by Friguls et al. [13]. N/A, not available. *: nonsense mutation

To date, infection may be related to the early onset diabetes. Our patient was hospitalized for refuse milk, drowsiness and fever. Laboratory results also suggest the presence of infection. After anti-infection and other symptomatic treatments, the symptoms of hyperglycemia and hyperlipidemia can be easily controlled and improved. Qin et al. also reported a 3-month-old boy occurred diarrhea with diabetes 1 month prior to the diagnosis of CGL. We speculate that the abnormal lipid metabolism caused by CGL will cause abnormal immune function and aggravate the symptoms of infection. Patients with CGL should be followed up closely to avoid acute infections.

There is no cure for CGL currently. Therapies to treat individuals with CGL include drug therapy, diet control, prevention of complications, and rehabilitation therapy. These treatments are mainly based on blood glucose and blood lipid control. Early intervention were used to reduce diabetes-related complications and cardiovascular risks. According to the therapeutical recommendations, our patient was treated with insulin and fed with deeply hydrolyzed formula, leading to FBG in normal ranges and a prompt decline of serum triglycerides level. The patient found slow weight gain and liver dysfunction at 5 months of age. In conclusion, our case reminds the importance of early identification, genetic analysis, and symptomatic treatment in the CGL, which are essential for improving the prognosis of children. Subsequent close follow-up of hepatic metabolic function, abdominal and cardiac ultrasound and intellectual development are necessary.

## Data Availability

Clinical data from the corresponding author will be available upon request.

## Abbreviations

CES: Clinical exome sequencing
CGL: Congenital generalized lipodystrophy
CRP: C reactive protein
FBG: Fasting blood-glucose
HGMD: Human Gene Mutation Database
IR: Insulin resistance
NICU: Neonatal intensive care unit
TG: Triglyceride
WBC: White blood cells
